# **Reducing the Power of the Alcohol Industry in Trade and Investment Agreement Negotiations Through Improved Global Governance of Alcohol** Comment on "What Generates Attention to Health in Trade Policy-Making? Lessons From Success in Tobacco Control and Access to Medicines: A Qualitative Study of Australia and the (Comprehensive and Progressive) Trans-Pacific Partnership"

**DOI:** 10.34172/ijhpm.2021.10

**Published:** 2021-02-17

**Authors:** Paula O’Brien

**Affiliations:** Melbourne Law School, The University of Melbourne, Melbourne, VIC, Australia.

**Keywords:** Law, Trade Policy, Global Governance, Alcohol, Industry, World Health Organization

## Abstract

The power of the alcohol industry pervades the global governance of alcohol. The influence of the industry is seen in trade and investment treaty negotiations, operating through direct and indirect means. Curbing the influence of the industry is vital to improving the treatment of health issues generally and in trade and investment policy particularly. The World Health Organization (WHO) has an opportunity to start to rein in the power of the industry with its current work on drafting an ‘action plan’ for 2022-2030 to implement the Global Strategy to Reduce the Harmful Use of Alcohol. The WHO working paper, however, proposes inadequate controls on alcohol industry influence. The WHO proposes ‘dialogue’ with the industry and allows the industry to take a role with government in public health labelling of alcohol. The public’s health will suffer if the WHO does not take a firmer stand against the industry in the ‘action plan.’

 The dominance of export industry interests in trade and investment agreement negotiations was elucidated by the insightful research by Townsend et al^1^ into the range of factors which were influential in determining the attention given to health concerns by Australia in the negotiation of the *Comprehensive and Progressive Trans-Pacific Partnership Agreement *(TPP).^2^ Through 25 interviews with politicians, public servants, industry representatives, public health policy advocates and academics, the research identified 16 conditions which influenced the treatment of public health concerns about tobacco, alcohol, nutrition and access to medicines in the negotiation of the TPP. The research found that the relatively positive public health outcomes for tobacco and access to medicines in the TPP negotiations were attributable to factors such as the strength of ministerial (trade and health), party political and public support for the health issue in question, the high quality of the evidence base, the existence of a relevant health treaty, and the presence of domestic legislation relating to the health matter. The poor outcomes from the TPP for alcohol and food were, in turn, due to the weakness or absence of these factors in relation to alcohol and food, coupled with the strength of exporter interests, the lack of knowledge about the particular health problem in the TPP negotiations, the lack of established advocacy networks, and the clash between the public health approach and the dominant market framing of the issue.^2^

 On the one hand, the Townsend et al finding about the significance of export industry interests to trade and investment agreements was not surprising as such agreements are principally intended to serve a country’s export interests. On the other hand, the research by Townsend et al revealed that industry interests were not just one amongst a myriad of factors which are weighed and balanced equally in these negotiations. Rather, a number of the conditions identified by Townsend et al as influencing whether health is given attention in the negotiations are subject to influence by industry. For example, the factor of public support for the health issue does not, on its face, appear to be about the industry, but industry activity outside of trade and investment negotiations, shapes public understanding of health risks. It also influences other Townsend et al factors, such as political support for health interventions, the presence of domestic regulation of health issues, and the state’s willingness to support greater global governance of the health issue. In this way, Townsend and colleagues’ parsing of the factors shaping the TPP negotiations brings transparency to the way in which the industry was able to dominate the conditions for negotiating the TPP – and, arguably, trade and investment agreements in general.

 The treatment of alcohol in the TPP makes clear how ubiquitous is the influence of the alcohol industry and the strength of its position in trade and investment agreements. In the TPP, parties agreed to a novel set of provisions around the supplementary labelling of wine and spirits,^3^which Australia has subsequently rolled out into several bilateral trade and investment agreements.^4^These provisions favour the alcohol industry and are potentially detrimental to public health, as they require that the industry be allowed to apply government-mandated information (including health information) on a label which is separate – or supplementary – to the original front or back label. This could mean that health warnings appear on a sticker which is ‘squeezed into’ some obscure place on the alcoholic beverage container and which therefore lacks the prominence necessary to be effective in communicating vital health information to the consumer. This is problematic as alcohol is a product that is causally connected to more than 200 diseases, conditions and injuries^5^(with at least 25 other diseases and conditions entirely attributable to alcohol)^6^and which caused 3 million deaths (5.3% of all deaths) and 132.6 million disability-adjusted life years (5.1% of all disability-adjusted life years) worldwide in 2016.^7^

 As noted by Townsend et al, there are strong alcohol exporter interests in Australia, the industry has cultivated good relationships with the government and opposition political parties, and the Minister for Trade is supportive of the alcohol industry. However, the alcohol industry has also actively worked in the domestic policy space to shape public and political views about the risks from drinking and the need for controls on alcohol. The industry seeks to control the ‘information environment’ to propagate ideas about alcohol as an ordinary commodity, which the majority can enjoy as an everyday part of a happy life, and as being only harmful to the ‘sorry few’ who drink irresponsibly.^8^The industry also suppresses health risk information.^9^These actions have multiple benefits for the industry: they create willing consumers for their products; they contribute to reluctance to impose regulatory controls on alcohol (at both the domestic and international levels); and they create a view of the alcohol industry as an important economic actor and alcohol as a legitimate domestic export, without regard for the health harms which flow from the product, especially in new markets^10^ where an increase in consumption and harm is expected.^11^

 Curbing the influence of the alcohol industry is therefore critical to many different efforts to improve the public’s health. Townsend et al make important suggestions for ways to limit the power of the industry in trade and investment agreement negotiations. At present, the place of the alcohol industry in global health governance is also under contest in the World Health Organization (WHO), with the outcome of this contest having implications for alcohol policy at the global and domestic levels, including trade and investment agreements. The WHO is developing ‘an action plan’^12^to strengthen the implementation of the Global Strategy to Reduce the Harmful Use of Alcohol(Global Alcohol Strategy).^13^ The action plan will include recommendations about the global and domestic initiatives which should be adopted to reduce alcohol consumption and related harm. The WHO’s Director-General will present the draft action plan to the WHO Executive Board for approval in January-February 2022.^12^ A first step in the development of the plan has been the publication by the WHO of a working paper,^7^ the consultation around which will inform multiple drafts of the action plan to be released for further comment in 2021.^12^

 The decision to embark on the creation of an action plan comes after a failed proposal by some (generally non-alcohol exporting) low- and middle-income countries to the WHO Executive Board to establish a working group to ‘review and propose the feasibility of developing an international instrument for alcohol control.’^14^The aim was for the working group to take steps towards the negotiation of a treaty on alcohol – a Framework Convention on Alcohol Control, modelled on the Framework Convention on Tobacco Control (FCTC).^15^ As found in Townsend et al, the existence of a treaty on the health issue of concern was crucial to the TPP negotiations, and the absence of a treaty on alcohol control was a deficit for public health, not filled by the Global Alcohol Strategy. The WHO Executive Board’s decision not to pursue the proposal for a working group and to instead opt for ‘an action plan’ is seen as a missed opportunity for the global governance of alcohol.^16^However, the process of creating an action plan can also be seen as a chance to lay the groundwork for the negotiation of a WHO treaty on alcohol in the future. This, as the FCTC was for tobacco and the WHO Doha Declaration on Trade-Related Aspects of Intellectual Property Rights and Public Health was for medicines in the TPP negotiations, would strengthen the public health position on alcohol in future negotiating rounds.

 The exclusion of the alcohol industry creates the best conditions for the WHO creating an action plan on alcohol that is effective in achieving the proximate goal of the plan of reducing alcohol-related consumption and harm. It also has the potential to shift the power of the industry more generally in the WHO, which is vital to building the pathway to an alcohol treaty. But, in my view, removing the alcohol industry from the WHO has more far-reaching consequences at the global and domestic level, including for trade and investment policy. One of the most important contributions made by the FCTC has been to delegitimise the global tobacco industry.^17^This diminution in the tobacco industry’s standing and power lessens its influence in global and domestic policy-making processes, including over the factors influencing trade and investment treaty negotiations as identified by Townsend et al. The inclusion of the ‘tobacco carve-out’ from the investment chapter of the TPP speaks to the parties’ sidelining of the tobacco industry, a position which is formally reflected in article 5.3 of the FCTC, which states that, ‘in setting and implementing their public health policies with respect to tobacco control, Parties shall act to protect these policies from commercial and other vested interests of the tobacco industry in accordance with national law.’^18 19^

 Unfortunately, the WHO has, to date, not adopted the same approach to the alcohol industry as it has for the tobacco industry.The working paper issued by the WHO to initiate the development of the action plan on alcohol envisages ‘dialogue’ between the WHO Secretariat and economic actors ‘on how they can best contribute to the reduction of alcohol-related harm within their core roles.’^7^It is also proposed that economic actors ‘ensure, within co-regulatory frameworks, the availability of easily-understood consumer information on the labels of alcoholic beverages (including composition, age limits, health warning and contraindications for alcohol use),’^7^suggesting that it is appropriate for states to allow the alcohol industry to determine aspects of public health labelling. This has not been a successful strategy in the past in Australia.^20^The WHO’s attempts to remove the industry from the action plan are, so far, inadequate, although it has ‘invited’ the industry to ‘abstain from interfering with alcohol policy development and evaluation.’^7^There is strong civil society opposition to the WHO proposal regarding the role of industry, including from the Global Alcohol Policy Alliance, which argues^21^:

 “*[A] lcohol industry entities (producers, distributors, retailers, etc ) are listed [in the WHO working paper] as stakeholders in equal standing alongside civil society and other UN [United Nations] organisations. This is inappropriate, given their inherent conflict of interest and long record of influence undermining effective alcohol policies, including in low- and middle-income countries... The alcohol industry should, instead, be addressed in a separate section with due regard to conflict of interest toward safeguarding public health.*”

 The WHO is the lead health agency in the UN system. The power and influence which it accords to the alcohol industry has repercussions across global health governance, including in the World Trade Organization, in regional and bilateral trade and investment treaties, and at the domestic level for each WHO member state. The TPP was a chapter in a long history of alcohol industry political and legal dominance at the international level.^22^The WHO’s action plan on alcohol, both in terms of the process followed to create the plan and the content ultimately included in the plan, represent an opportunity for the WHO to start reining in the power of the alcohol industry. By removing the cloak of legitimacy from the alcohol industry, the WHO would make a major contribution to weakening the control of the alcohol industry over many of the factors which Townsend et al show are significant, and in enabling concerns about alcohol’s impacts on the public health to assume a more central position in domestic policy, including in trade and investment agreement negotiations.

## Acknowledgements

 I acknowledge the assistance of my research assistant, Eliza Waters.

## Ethical issues

 Not applicable.

## Competing interests

 Author declares that she has no competing interests.

## Author’s contribution

 PO is the single author of the paper.
